# In silico genomic insights into bacteriophages infecting ESBL-producing *Escherichia coli* from human, animal, and environmental sources

**DOI:** 10.1186/s12866-025-03913-9

**Published:** 2025-04-08

**Authors:** Mabel Kamweli Aworh, Opeyemi U. Lawal, Beverly Egyir, Rene S. Hendriksen

**Affiliations:** 1 Department of Biological and Forensic Sciences, Fayetteville State University, Fayetteville, NC USA; 2 ECU Brody School of Medicine, Department of Public Health, East Carolina University, Greenville, NC USA; 3 School of the Environment, University of Windsor, Windsor, ON Canada; 4 Canadian Research Institute for Food Safety, Department of Food Safety, University of Guelph, Guelph, ON Canada; 5 Department of Bacteriology, College of Health Sciences, Noguchi Memorial Institute for Medical Research, University of Ghana, Accra, Ghana; 6 Technical University of Denmark, National Food Institute, WHO Collaborating Centre (WHO CC) for Antimicrobial Resistance in Foodborne Pathogens and Genomics, FAO Reference Laboratory (FAO RL) for Antimicrobial Resistance, European Union Reference Laboratory for Antimicrobial Resistance (EURL-AMR), Kongens Lyngby, Denmark

**Keywords:** *Escherichia coli*, Prophage, Bacteriophages, Abattoir workers, Cattle, Abattoir environment, One Health, Antimicrobial resistance, Nigeria

## Abstract

**Background:**

The emergence of antimicrobial resistance (AMR) in *Escherichia coli*, particularly extended-spectrum beta-lactamase-producing *E. coli* (ESBL-EC), is a global public health concern. Bacteriophages (phages) play a significant role in bacterial evolution and the spread of antibiotic resistance genes (ARGs). This study investigates prophages integrated within ESBL-EC genomes to assess their diversity, gene content, and potential contributions to ESBL-EC persistence across human, animal, and environmental reservoirs. Between May and December 2020, a cross-sectional study was conducted in Abuja and Lagos, collecting 448 stool, cecal, and environmental samples from abattoir workers, slaughtered cattle, and the abattoir environment. ESBL-EC genomes from these samples, obtained in an earlier study, were analyzed for phage regions using PHASTER. Intact prophages were analyzed in silico using computational tools to detect ARGs, ESBL genes, virulence factors, and heavy metal resistance. Their genomic relationships were examined with statistical significance of *p* < 0.05.

**Results:**

Out of 448* s*amples, ESBL-EC prevalence was 21.7% (97/448). Among *97* ESBL-EC isolates, 646 prophage regions were detected, with 30% (194/646) classified as intact phages. Among the 158 phages with genus assignments, *Punavirus* was the most prevalent (60.1%). *Escherichia* was the most frequent predicted host (308/646), particularly in cattle (*n* = 143) and human (*n* = 124) sources. Among ESBL-EC genomes, 83.5% (81/97) with intact phages carried phage-associated ARGs, 76.3% (74/97) carried phage-associated ESBL genes, 18.6% (18/97) harbored phage-associated virulence factors, 15.5% (15/97) contained phage-associated plasmids, and 10.3% (10/97) had heavy metal resistance. The most prevalent phage-associated ARGs detected were *qnrS1* (73/81) and *bla*_*CTX-M-15*_* (72/81)*. Two isolates recovered from abattoir workers carried two phage-like plasmids, each harboring either *tet(A)* or *bla*_*CTX-M-55*_ gene. The predominant phage lifestyles were temperate (*n* = 182), mainly in the *Peduoviridae* family, and lytic (*n* = 12) in the *Punavirus* genus.

**Conclusion:**

This is the first study in Nigeria to characterize phages in ESBL-EC isolates at the One Health interface. The presence of intact phages in humans, animals, and the environment underscores the complex interactions shaping phage ecology. The discovery of ARGs, virulence genes, and heavy metal resistance within prophages suggests a potential role in AMR dissemination. Future research should focus on elucidating mechanisms of ARG transfer mediated by phages in One Health settings.

**Supplementary Information:**

The online version contains supplementary material available at 10.1186/s12866-025-03913-9.

## Introduction

The global burden of antimicrobial resistance (AMR) poses a significant threat to human, animal, and environmental health [1, 2]. Among AMR pathogens, extended-spectrum beta-lactamase (ESBL)-producing *Escherichia coli* (ESBL-EC) has emerged as a critical concern due to its ability to hydrolyze 3rd- and 4th-generation cephalosporins, severely limiting treatment options for Gram-negative bacterial infections [3–5]. The production of ESBL enzymes, often encoded by plasmid-mediated resistance genes such as *bla*_*CTX-M*_ and *bla*_*TEM*_, has facilitated the rapid dissemination of ESBL-EC across human, animal, and environmental reservoirs, contributing to rising morbidity, mortality, and healthcare costs [6, 7].

Traditionally, ESBL-EC was associated with healthcare settings. However, reports of community-acquired ESBL-EC infections have increased in recent years [3, 8–10]. Food-producing animals, particularly cattle, and their associated environments have been identified as important reservoirs for ESBL-EC [3]. These bacteria can be transmitted to humans through direct occupational exposure, such as abattoir work, or indirectly via the food chain and contaminated environments [3, 11, 12]. In sub–Saharan Africa and countries like Nigeria, where cattle farming and beef consumption are integral to livelihoods and diets, the potential for ESBL-EC dissemination along the food chain is particularly concerning [13].

Bacteriophages (phages), viruses that specifically infect bacteria, play a key role in bacterial evolution, diversity, and adaptation [14, 15]. Prophages, which are phage genomes integrated into bacterial chromosomes, are known to contribute to horizontal gene transfer, including the spread of antimicrobial resistance genes (ARGs) and virulence factors [15, 16]. The presence of ESBL genes is a significant marker of antibiotic resistance, and these genes can be acquired through horizontal gene transfer (HGT), including through plasmids, transposons, and possibly even phages [3, 17, 18]. While most studies on ESBL-EC have focused on plasmid-mediated resistance, the contribution of prophages to the genetic diversity, pathogenicity, and resistance mechanisms of ESBL-EC remains underexplored [17, 18]. Characterizing bacteriophages associated with ESBL-EC may provide insights into their role in AMR dissemination and the evolution of pathogenic bacteria in various reservoirs [17–19].

The abattoir ecosystem, which includes slaughtered cattle, workers, and the surrounding environment, represents a critical interface where zoonotic pathogens such as ESBL-EC may circulate and evolve [3, 20]. Abattoir workers, through occupational exposure, face a heightened risk of colonization or infection with resistant bacteria, while improper waste management can facilitate the environmental dissemination of ESBL-EC [3]. Furthermore, phages associated with ESBL-EC in this ecosystem may carry genes that contribute to bacterial survival, virulence, and resistance under selective pressures imposed by antimicrobial use in livestock production [17, 19].

Here, we hypothesize that ESBL-EC isolated from beef cattle processing chain carry diverse prophages capable of encoding virulence factors and facilitating the transmission of resistance genes along the food chain. Using bioinformatics approaches, this study investigated the prophage content of whole genome sequenced ESBL-EC isolated from abattoir workers, beef cattle and abattoir environments to better understand their potential roles in AMR dissemination and pathogen evolution. By investigating the interplay between ESBL-EC and their associated phages in the abattoir ecosystem, this study seeks to contribute to a better understanding of the mechanisms underlying AMR dissemination and to identify potential phage-based strategies for controlling resistant bacteria.

This study aims to identify and classify prophages integrated within ESBL-EC genomes, assess the diversity and distribution of prophage-encoded genes, including those associated with virulence and ARGs, and explore the potential role of bacteriophages in the evolution and dissemination of ESBL-EC across human, animal, and environmental reservoirs.

## Methods

### Study design and sample collection

In a previous cross-sectional study conducted between May and December 2020 in two large abattoirs in Abuja and Lagos, Nigeria, we collected a total of 448 samples, with one abattoir sampled per city. Stool samples were obtained from randomly selected abattoir workers who provided written informed consent prior to enrollment in the study. Cecal contents from slaughtered cattle were aseptically collected directly from the cecum using sterile universal containers. Samples were collected from the abattoir environment, including lairage litter and abattoir wastewater. All samples were transported in ice to the Nigeria Centre for Disease Control’s National Reference Laboratory, Abuja, where they were analyzed for the presence of ESBL-EC as previously described [3].

### Isolation and identification of ESBL-EC Isolates

The isolates from the previous study were identified using a modified version of the World Health Organization's ESBL-Tricycle project protocol [21]. Briefly, 1 g of stool, 1 g of cecal content, and 30 g of lairage litter were enriched in buffered peptone water at a ratio of 1:10 and incubated at 37 °C for 24 h. A 10-µl loopful of the overnight enrichment culture was then streaked onto MacConkey agar supplemented with 1 mg/L cefotaxime and incubated at 37 °C for 24 h. Colonies with morphological characteristics indicative of *E. coli* (pink) were streaked onto eosin methylene blue agar for further purification and incubated under the same conditions. Suspected ESBL-EC isolates were confirmed biochemically using the Microbact GNB 24E system (Oxoid, UK), following the manufacturer’s guidelines [3].

### Whole-genome sequencing of ESBL-EC

Whole genome sequencing of ESBL-EC genomes was performed under the Fleming funded SEQAFRICA Project at the Noguchi Memorial Institute for Medical Research, University of Ghana. Genomic DNA was extracted and purified from overnight cultures of the bacteria isolates using the Qiagen kit, following the manufacturer’s protocol. DNA concentrations were quantified using the Qubit 4.0 Fluorometer (Thermo Fisher Scientific, USA). Library preparation was carried out using the Nextera Flex kit and assessed for quality using the 2100 Bioanalyzer (Agilent) and the Kapa SYBR Fast qPCR Kit. The DNA libraries were pooled, and sequencing was performed using an Illumina MiSeq platform with a 2 × 300 bp paired-end configuration. The raw sequencing reads were processed using Trimmomatic for quality filtering (minimum Phred score ≥ 20), adapter removal, and length filtering. Quality assessment of the processed reads was conducted with FastQC, and high-quality reads were subsequently assembled de novo using the Unicycler assembler v0.4.9 [22]

### Analysis of phage regions and gene content

Genome analysis using in silico computational methods was performed on each ESBL-EC strain to identify and extract prophages. The detection and annotation of phage regions within bacterial genomes were performed with PHASTER [23] and PhaBox [24]. For each ESBL-EC isolate, complete, questionable, and incomplete prophage regions were identified based on PHASTER’s scoring system (intact: score > 90; questionable: score 70–90; incomplete: score < 70). Intact prophage regions were selected for downstream analysis.

Extracted prophage sequences were screened for genes encoding virulence factors, tailspike proteins, antibiotic resistance, and heavy metal resistance. Virulence genes were identified using the VirulenceFinder Database (VFDB) [25], while ARGs were detected using the Comprehensive Antibiotic Resistance Database (CARD) [26]. To identify genes associated with tailspike proteins and heavy metal resistance, BLASTn and BLASTp searches were conducted against TSPDB [27] and Bacmet [28] custom databases, respectively.

### Detection of phage-plasmid elements

To identify phage-plasmid hybrid elements, sequences were analyzed using tools such as PlasmidFinder [29] and MOB-suite [30]. Additionally, prophage regions were compared with known plasmid sequences using BLASTn to confirm potential integrations. Specific structural features, such as integrase and transposase genes, were further examined to validate phage-plasmid recombination events.

### Phylogenetic analysis of phages

All extracted prophage sequences were aligned using MAFFT v7, and the alignment was refined to remove poorly aligned regions using Gblocks. A maximum-likelihood phylogenetic tree was constructed with RAxML v8.2.12 using the GTR + G model of nucleotide substitution and 1,000 bootstrap replicates to assess branch support. To correlate phage lineages with gene content, a heatmap showing the presence or absence of phage hallmark genes was generated in iTOL v6 [31].

### Statistical analysis

All statistical analyses were performed using R statistical package version 4.4.2. Phage diversity across the different sample sources (human, animal, and environmental) was assessed. Additionally, the distribution of prophage-encoded ARGs and virulence factors across sample sources was compared using chi-square tests, as appropriate. A multinomial logistic regression model was used to evaluate the association between phage-related genes and the likelihood of a sample being categorized as originating from different sources. Statistical significance was set at a *p*-value < 0.05. Data visualization was performed using the ggplot2 package for comprehensive graphical representation of results. 

## Results

Two sizable abattoirs in Abuja (*n* = 228) and Lagos (*n* = 220) yielded a total of 448 samples, including human stool (*n* = 118), cattle cecal content (*n* = 272), and abattoir environment samples (*n* = 58). Of these 97 ESBL-EC isolates were obtained and whole genome sequenced comprising beef cattle (*n* = 44, 45.4%), abattoir workers (*n* = 40, 41.2%) and abattoir environment (*n* = 13, 13.4%).

In silico phage analysis detected a total of 646 prophage regions, of which 194 (30%) were intact phages, 340 (52.6%) were incomplete, while 112 (17.3%) were questionable. The prevalence of intact phages originating from abattoir workers was 43.8% (*n* = 85), followed by 43.3% (*n* = 84) from beef cattle and 12.9% (*n* = 25) from abattoir environment. There was a statistically significant difference in the distribution of intact phages across the three sources (*X*^*2*^ = 36.5; *df* = 2, *p* < 0.001). More intact phages were detected in samples originating from Lagos (*n* = 109; 56.2%) when compared to Abuja (*n* = 85; 43.8%). The prevalence of intact phages across these sources was statistically different between the two abattoirs (*X*^*2*^ = 59.0; *df* = 2, *p* < 0.001).

### Distribution of phages detected in ESBL-EC isolates across different sources for four phage categories

**Fig. 1:**
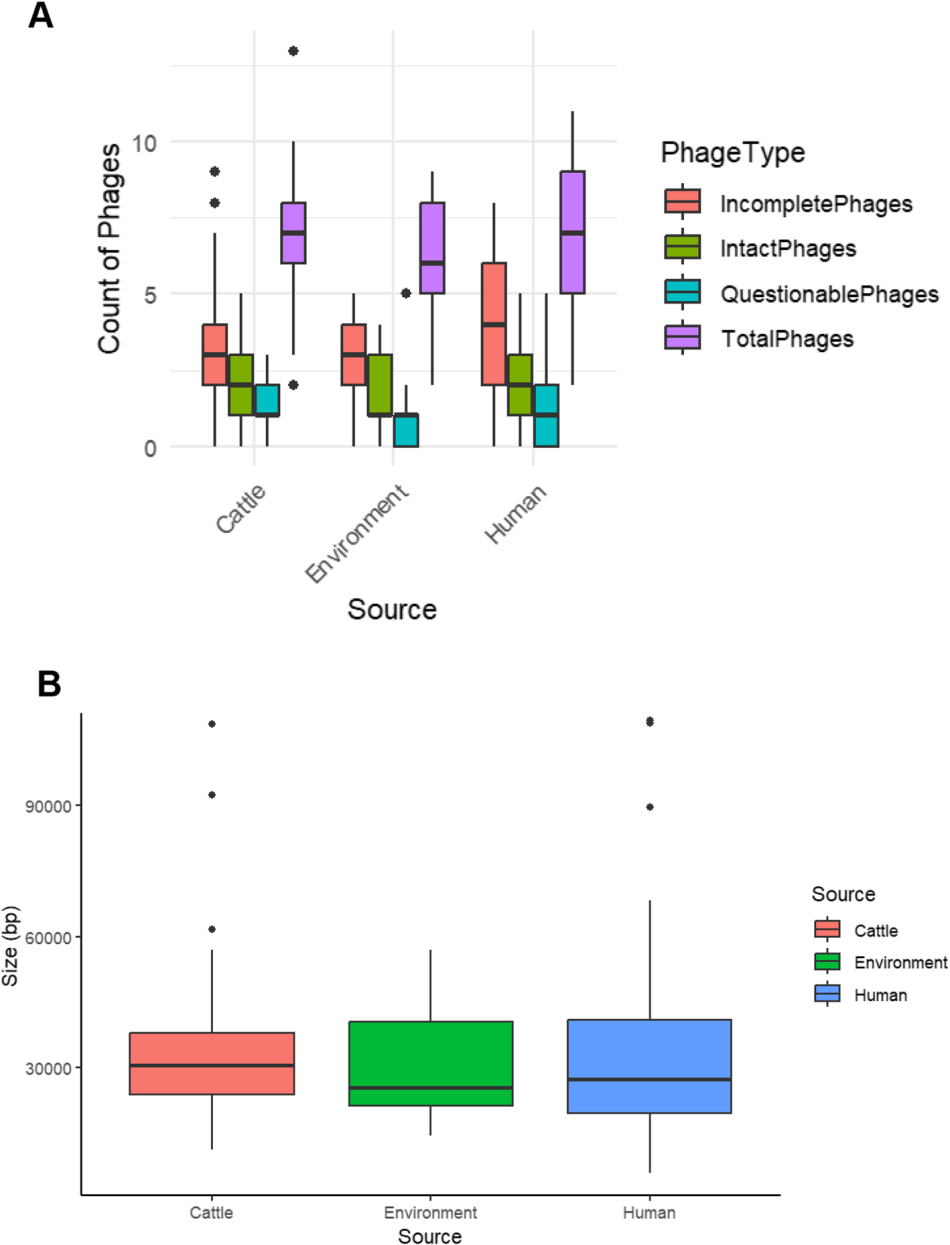
**A** Distribution of phage regions detected in ESBL *E. coli* isolated from abattoir workers, beef cattle and abattoir environment. Boxplot showing the count of phage regions across three sources: cattle, environment, and human. The three phage regions analyzed are incomplete phages, intact phages, and questionable phages, while total phages is the sum of the phage regions observed. The boxes represent the interquartile range (IQR), with the median indicated by the horizontal line within each box. Whiskers extend to the smallest and largest values within 1.5 times the IQR, while dots represent outliers. Total phages show the highest counts and variability across all sources, particularly in human and cattle samples. Environmental samples exhibit consistently lower phage counts across all categories". **B** Distribution of intact phage genome sizes in ESBL-EC phages across all sources

The distribution of phages across beef cattle, abattoir environments and abattoir workers were analyzed using four phage categories: incomplete phages, intact phages, questionable phages, and total phages (Fig. 1A). The boxplot shows that human samples demonstrated the widest range of total phages, with several outliers indicating higher counts in certain samples. In contrast, samples originating from the abattoir environment had the lowest overall phage counts across all categories, with narrower interquartile ranges. Notably, outliers were observed for total phages in cattle and human samples, suggesting variability in phage counts within these sources.

Intact phages showed consistent patterns across sources, with counts higher than incomplete phages and questionable phages. Questionable phages were the least represented phage type, showing minimal variation and consistently lower medians across all sources.

### Phage region size distribution across different sources

The summary of the distribution of intact phage genome sizes from beef cattle, abattoir environment, and abattoir workers from the two abattoirs is shown in Fig. 1B. The median phage genome size is consistent across all three sources, falling within the range of approximately 30 kb with interquartile ranges spanning similar lengths across the three sources. However, there is notable variability in genome sizes of intact phages from abattoir workers, as evidenced by a wider interquartile range and the presence of outliers extending beyond 90 kb.

### Diversity and prevalence of hallmark genes in analyzed phage regions

The *Caudoviricetes class* and *Peduoviridae family* were the most prevalent among the phages identified in this study, which were sourced from human, animal, and environmental samples. These phages are well-known for their ecological significance and widespread presence, frequently infecting a diverse range of bacterial hosts. Of 646 observations, 488 (75.6%) lacked genus-level assignments. Among the 158 phages with genus assignments, *Punavirus* was the most prevalent genus within the class *Caudoviricetes*, accounting for 95 instances (60.1%), while *Pedovirus* is more common in the *Peduoviridae* family with 14 instances (8.9%). In terms of lifestyle, temperate intact phages were predominant (182/194), primarily in the *Peduoviridae* family. In contrast, lytic intact phages were less common (12/194) and were predominantly found in the *Punavirus* genus of the *Caudoviricetes* class.

**Fig. 2:**
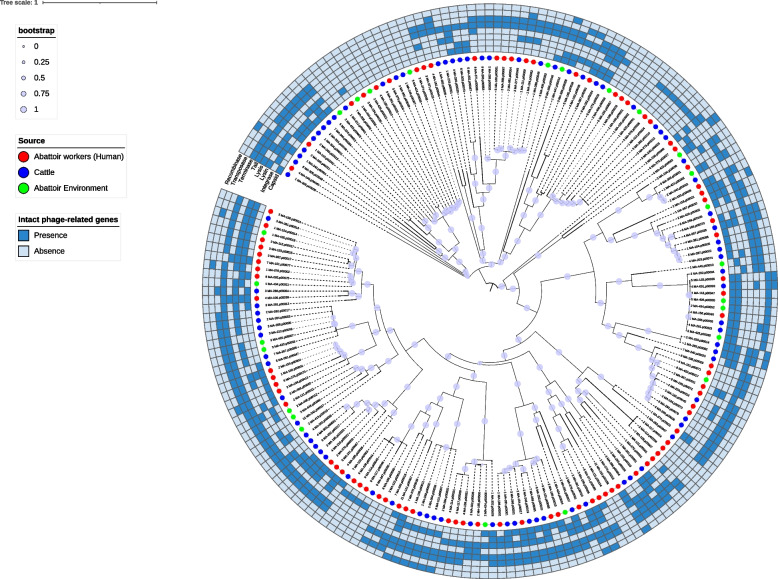
Phylogenetic tree depicting the distribution of hallmark genes among phages detected in ESBL-EC isolates. Prophage sequences were aligned using MAFFT v7, and a maximum likelihood tree was constructed with RAxML v8.2.12 using the GTR + G model of nucleotide substitution and 1,000 bootstrap replicates. Trees were mapped to the presence or absence of phage hallmark genes and visualized using iTOL v6

The most frequently detected intact phage-associated hallmark genes were *tail* (*n* = 155; 79.9%), *terminase* (*n* = 117; 60.3%), *integrase* (*n* = 85; 43.8%), *lysin* (*n* = 82; 42.3%), and *capsid* (*n* = 78; 40.2%), highlighting their essential roles in key phage lifecycle processes such as structural assembly, genome packaging, integration into host genomes, and host cell lysis (Fig. 2). Less frequently detected genes included *transposase* (*n* = 22; 11.3%), *recombinase* (*n* = 17; 8.8%), and *lysis* (*n* = 16; 8.2%), suggesting that while these genes may contribute to genetic mobility and host interaction, they are less predominant in the analyzed phage population.

### Phage host prediction confidence

**Fig. 3:**
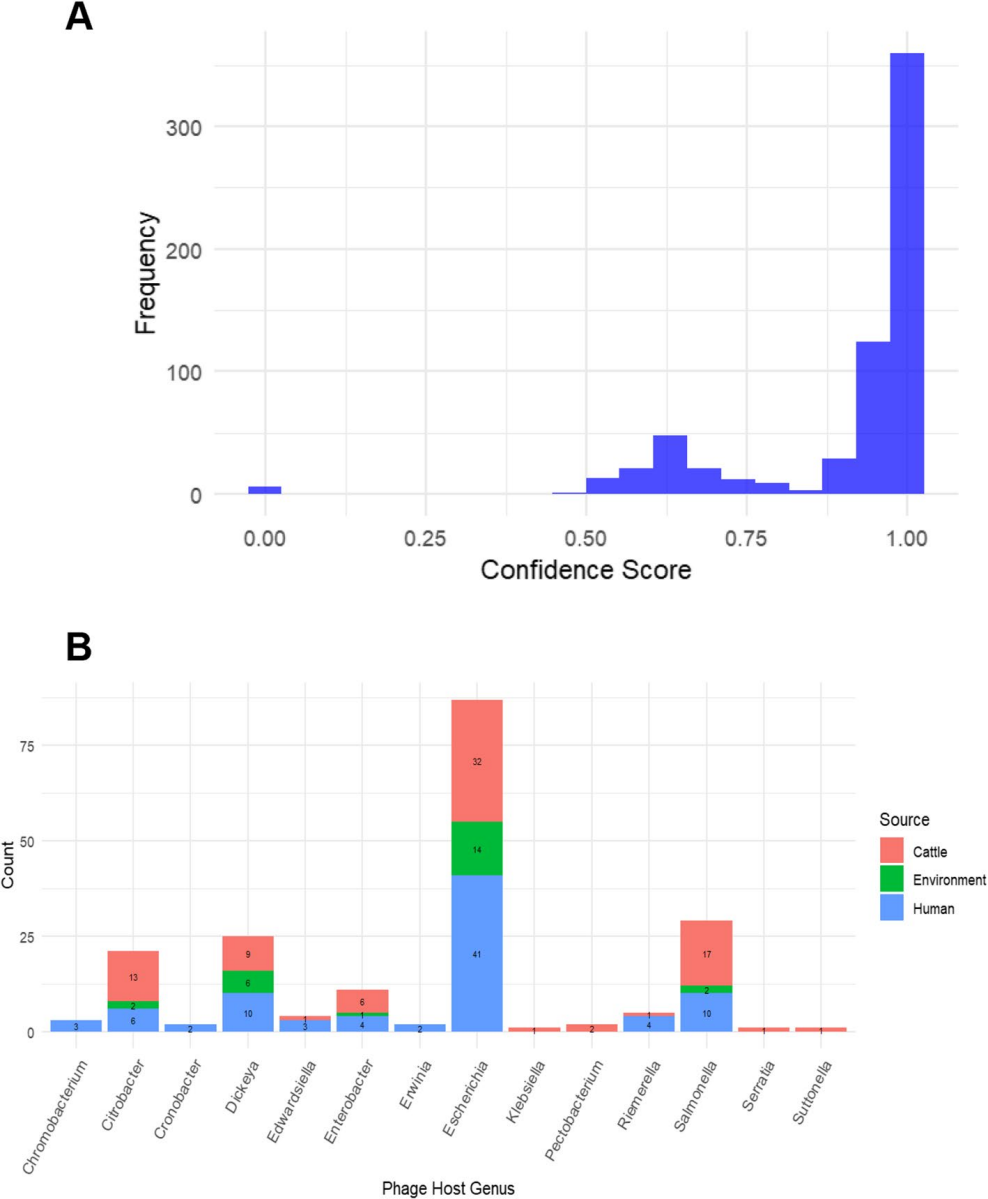
**A** Distribution of Confidence Scores for Phage Host Prediction. The histogram illustrates the distribution of confidence scores for phage host prediction. Most phages exhibit a high confidence score close to 1.0, suggesting strong certainty in host prediction accuracy. A smaller subset of phages shows intermediate confidence scores between 0.5 and 0.9, while very few phages have low confidence scores close to 0.0. **B** Distribution of Phage Host Genus by Source. The bar chart represents the distribution of phage host genera across different sources: cattle (red), environment (green), and human (blue). The y-axis indicates the count of phage host occurrences, while the x-axis lists the identified host genera. The stacked bars illustrate the relative contribution of each source to the total count for each genus

The confidence scores for phage host prediction are presented in Fig. 3A. Most phages (*n* > 300) were assigned a confidence score of 1.0, reflecting a high level of certainty in host prediction. A smaller proportion of phages displayed intermediate confidence scores ranging between 0.5 and 0.9, suggesting some uncertainty in the prediction. Only a negligible number of phages exhibited low confidence scores (< 0.25), indicating minimal prediction reliability. These results demonstrate that the phage host prediction method used in this study was highly accurate for most analyzed phages, with only a limited subset requiring further investigation.

### Phage host prediction

Among intact phages, the distribution of predicted phage host genera across different sources (cattle, environment, and human) revealed a significant dominance of *Escherichia* (*n* = 87), which was primarily associated with human (*n* = 41) and cattle (*n* = 32) sources (Fig. 3B). Other frequently identified genera included *Salmonella, Dickeya* and *Citrobacter* though their presence varied across sources. The beef cattle samples displayed a more diverse range of host genera but with relatively lower counts. The stacked bar plot also highlights that some genera, such as *Enterobacter* and *Edwardsiella* were primarily associated with cattle and human sources, respectively. Overall, this analysis suggests host-genus specificity among different sources, with notable overlaps in some genera.

### Phage-associated resistance determinants by source: ARGs, ESBL genes, virulence genes and heavy metal resistance

**Fig. 4:**
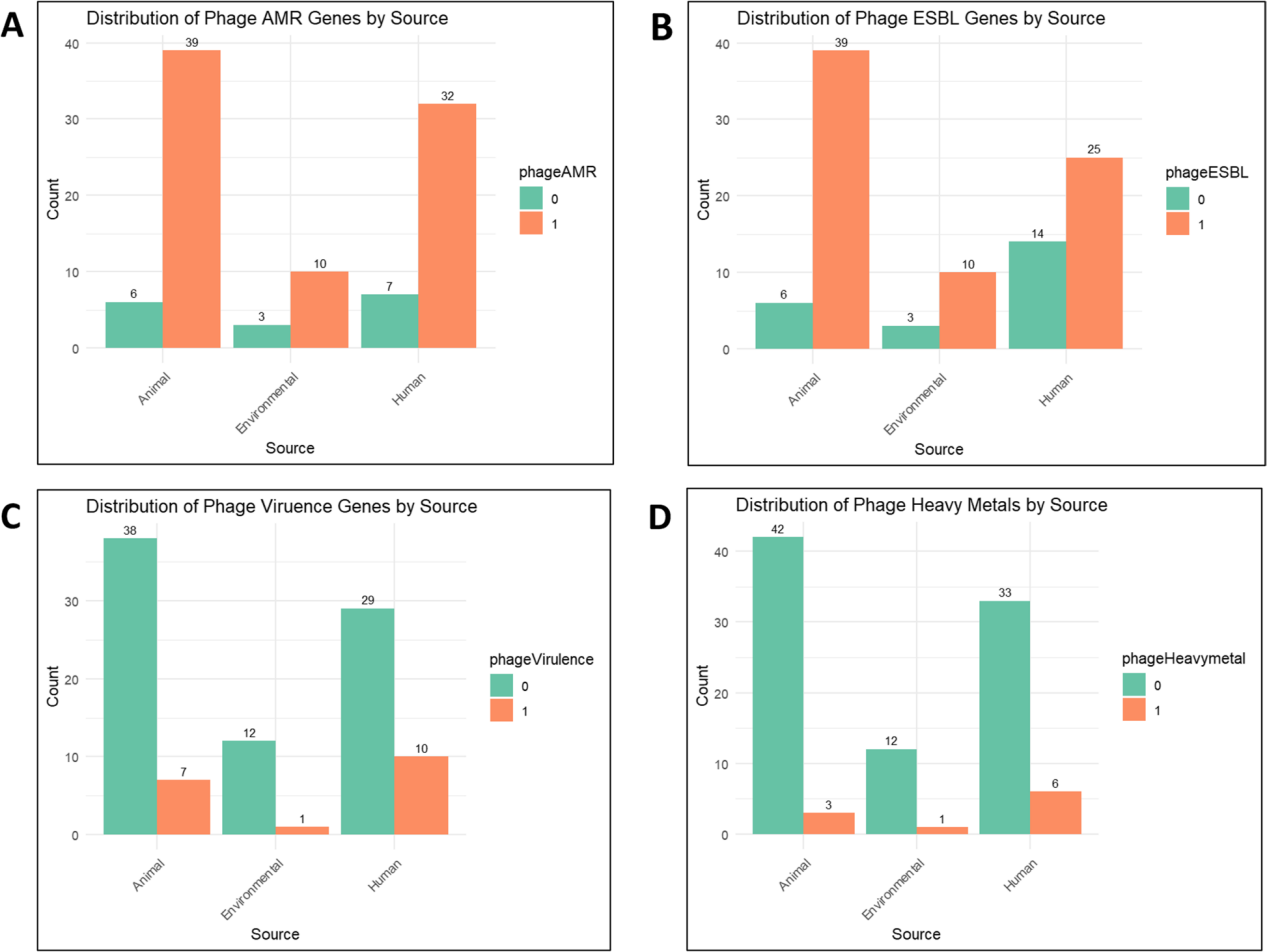
A-D: Phage-Associated Resistance Determinants by Source: ARGs, ESBL Genes, Virulence Genes and Heavy-Metal Resistance. This figure illustrates the distribution of AMR genes, ESBL genes, Virulence genes and Heavy metal resistance in phages across different sources: beef cattle, abattoir environment, and abattoir workers. In the figure legends, "0" indicates the absence of the characterized variable, while "1" denotes its presence

- Phage-associated AMR genes by source
  Out of 97 ESBL-EC isolates, 81 isolates (81/97) containing intact phages carried phage-associated ARGs. Among the phages analyzed, those originating from beef cattle exhibited the highest frequency of ARGs (39/81), followed closely by phages from abattoir workers (32/81). Abattoir environmental sources showed a comparatively lower occurrence of ARGs (10/81) as shown in Fig. 4A. Notably, the absence of ARGs in phages was observed more frequently in human (*n* = 7) and animal (*n* = 6) sources, with environmental sources exhibiting the lowest count (*n* = 3). Further analysis of ARGs showed that the most prevalent resistance genes detected in the intact phages were associated with extended-spectrum beta-lactamases (91.4%; 74/81) and quinolones (91.4%; 74/81) as shown in Table 1.

**Table 1:** Phage-Associated antimicrobial resistance genes in ESBL *Escherichia coli* identified using CARD

AMR genes	AMR class	Overall *N* = 81 (%)	Abattoir workers *N* = 32 (%)	Beef Cattle *N* = 39 (%)	Abattoir environment *N* = 10 (%)
*qnrS1*	Quinolones	73 (90.1)	25 (78.1)	38 (97.4)	10 (100)
*qnrS4*		1 (1.2)	0 (0)	1 (2.6)	0 (0)
*blaCTX-M-15*	Beta-lactamases	72 (88.9)	24 (75)	38 (97.4)	10 (100)
*blaCTX-M-55*		2 (2.5)	1 (3.1)	1 (2.6)	0 (0)
*blaTEM-1*		15 (18.5)	7 (21.9)	4 (10.3)	4 (40)
*mdfA*	Efflux Pump	14 (17.3)	7 (21.9)	4 (10.3)	3 (30)
*emrE*		10 (12.3)	6 (18.8)	3 (7.7)	1 (10)
*qacl*		1 (1.2)	1 (3.1)	0 (0)	0 (0)
*aph(6)-ld*	Aminoglycosides Beta-lactamases	13 (16)	6 (18.8)	3 (7.7)	4 (40)
*aph(3’’)-lb*		13 (16)	6 (18.8)	3 (7.7)	4 (40)
*ant(3″)-lla*		1 (1.2)	1 (3.1)	0 (0)	0 (0)
*sul2*	Folate pathway antagonists	13 (16)	6 (18.8)	3 (7.7)	4 (40)
*sul3*		1 (1.2)	1 (3.1)	0 (0)	0 (0)
*dfrA1*		1 (1.2)	1 (3.1)	0 (0)	0 (0)
*dfrA14*		1 (1.2)	1 (3.1)	0 (0)	0 (0)
*dfrA17*		1 (1.2)	0 (0)	1 (2.6)	0 (0)
*tetA*	Tetracyclines	3 (3.7)	3 (9.4)	0 (0)	0 (0)
*baeS*	Two-Component Regulatory System	3 (3.7)	2 (6.3)	0 (0)	1 (10)
*baeR*		3 (3.7)	2 (6.3)	0 (0)	1 (10)
*cpxA*	Stress Response Regulator	2 (2.5)	1 (3.1)	0 (0)	1 (10)

*CARD* Comprehensive Antibiotics Resistance Database

b) Phage-associated ESBL genes by source
  Out of 97 ESBL-EC isolates, 74 isolates (74/97) containing intact phages carried phage-associated ESBL genes. Among the phages analyzed, those originating from beef cattle exhibited the highest frequency of ESBL genes (39/74), followed closely by phages from abattoir workers (25/74). Abattoir environmental sources showed a comparatively lower occurrence of ESBL genes (10/74) as shown in Fig. 4B. The most prevalent ESBL genes detected was *blaCTX-M-15* (*n* = 72; (37.1%) followed by *blaCTX-M-55* (*n* = 2; (2.5%) as shown in Table 1.
c) Phage-associated virulence genes by source
  Out of 97 ESBL-EC isolates, 18 isolates (18/97) containing intact phages carried phage-associated virulence genes. Among the phages analyzed, those originating from abattoir workers exhibited the highest frequency of virulence genes (10/18), followed closely by phages from beef cattle (7/18). Abattoir environmental sources showed a comparatively lower occurrence of virulence genes (1/18) as shown in Fig. 4C. Notably, the absence of virulence genes in phages was observed more frequently in beef cattle (*n* = 38) and abattoir workers (*n* = 29), with abattoir environmental sources exhibiting the lowest count (*n* = 12). A total of 26 phage-associated virulence genes were detected, with some isolates carrying multiple virulence genes (Table 2). The most prevalent virulent genes across humans, animals and the environment were *fimB* (*n* = 5, 19.2%) and *fimE* (*n* = 4, 15.4%).

**Table 2:** Phage-Associated virulence genes detected in ESBL *Escherichia coli* isolated from all sources identified using VFDB

Virulence genes	Overall *N* = 26 (%)	Abattoir Workers *N* = 13 (%)	Beef Cattle *N* = 11 (%)	Abattoir Environment *N* = 2 (%)
*gtrA*	5 (19.2)	2 (15.4)	3 (27.2)	0 (0)
*fimB*	5 (19.2)	2 (15.4)	2 (18.2)	1 (50)
*fimE*	4 (15.4)	1 (7.7)	2 (18.2)	1 (50)
*gndA*	1 (3.8)	0 (0)	1 (9.1)	0 (0)
*ugd*	4 (15.4)	3 (23.1)	1 (9.1)	0 (0)
*galF*	3 (11.5)	2 (15.4)	1 (9.1)	0 (0)
*KP1_RS17355*	1 (3.8)	0 (0)	1 (9.1)	0 (0)
*espL1*	3 (11.5)	3 (23.1)	0 (0)	0 (0)

*VFDB* Virulence Factor Database

d) Phage-associated heavy metal resistance by source
  Out of 97 ESBL-EC isolates, 10 isolates (10/97) containing intact phages carried phage-associated heavy metal resistance. Among the phages analyzed, those originating from abattoir workers exhibited the highest frequency of heavy metal resistance (6/10), followed closely by phages from beef cattle (3/10). Abattoir environmental sources showed a comparatively lower occurrence of heavy metal resistance (1/10) as shown in Fig. 4D. Notably, the absence of heavy metal resistance in phages was observed more frequently in beef cattle (*n* = 42) and abattoir workers (*n* = 33), with abattoir environmental sources exhibiting the lowest count (*n* = 12). The phage-associated heavy metal resistance gene *emrE* was detected in isolates from abattoir workers, beef cattle and abattoir environments.

### Phage-associated plasmids and tail spike proteins

Out of 97 ESBL-EC isolates, 15 isolates (15/97) containing intact phages carried phage-associated plasmids. Among the bacteriophage sequences analyzed, those originating from abattoir workers exhibited the highest frequency of heavy metal resistance (12/15), followed closely by phages from beef cattle (3/15). The most prevalent phage-associated plasmids identified in isolates from both human and cattle sources were IncY (*n* = 5) and IncFIB (H89-PhagePlasmid) (*n* = 4). Additionally, IncFIB(K) was detected exclusively in the cattle isolate, while IncR (*n* = 3), IncHI1B(pNDM-CIT) (*n* = 1), and p0111 were found only in human isolates (Additional file 1). Two isolates recovered from abattoir workers carried phage-like plasmids with lytic machinery, each harboring either the *tet(A)* or *bla*_*CTX-M-55*_ gene, with one isolate from each abattoir/city.

In this study, we identified a tail spike protein (FN543502.1_cds_CBG90373.1) in phages from ESBL-EC isolates obtained from both human and cattle sources, while another tail spike protein (UFWO01000001.1_cds_SUX74655.1) was detected exclusively in the same human sample. The tail spike protein is known for its role in bacteriophage-mediated bacterial infection, where it facilitates the adsorption of phages to bacterial cell surfaces. In the samples analyzed, the protein was present in one of human isolate and three of the cattle isolates, suggesting a genetic overlap between the bacterial populations from the two hosts (Additional file 1).

### Association between phage-related genes and source classification

A multinomial logistic regression model was used to evaluate the association between phage-related genes (*integrase*, *terminase*, *capsid*, *lysin*, and *tail*) and the likelihood of a sample originating from human or environmental sources. The model revealed that the Intercept for the human category was significant (β = 0.8497, 95% CI: 0.030–1.669, *p* = 0.042), suggesting that, in the absence of additional predictors, samples from human sources are significantly different from the reference category (beef cattle). However, none of the tested genes were statistically significant predictors of the source category (all *p*-values > 0.05).

Although *integrase genes* (β = 0.5990, *p* = 0.243 for environment; β = 0.3924, *p* = 0.257 for human) showed a positive trend, the association was not statistically significant. Similarly, *capsid*, *lysin*, and *tail genes* exhibited negative estimates across both human and environmental samples but did not reach statistical significance. The model had a residual deviance of 369.16 and an AIC of 393.16, indicating a moderate model fit (Table 3).

**Table 3:** Multinomial logistic regression results for predicting source category (environment, human)

Predictor	Estimate (β)—Environ	Std. Error	95% CI (Environ)	*p*-value (Environ)	Estimate (β)—Human	Std. Error	95% CI (Human)	*p*-value (Human)
Intercept	−0.1863	0.5270	(−1.219, 0.847)	0.724	0.8497	0.4182	(0.030, 1.669)	**0.042**
Integrase	0.5990	0.5133	(−0.407, 1.605)	0.243	0.3924	0.3461	(−0.286, 1.071)	0.257
Terminase	−0.3609	0.6126	(−1.562, 0.840)	0.556	−0.3727	0.4090	(−1.174, 0.429)	0.362
Capsid	−0.5967	0.5540	(−1.682, 0.489)	0.281	−0.3575	0.3609	(−1.065, 0.350)	0.322
Lysin	−0.4059	0.5403	(−1.465, 0.653)	0.453	−0.2455	0.3453	(−0.922, 0.431)	0.477
Tail	−0.8433	0.7429	(−2.299, 0.613)	0.256	−0.6280	0.5479	(−1.702, 0.446)	0.252

Statistically significant results (*p* < 0.05) are in **bold**

## Discussion

Bacteriophages are important participants in microbial ecosystems as they contribute to bacterial diversity, evolution, and adaptation through processes including horizontal gene transfer [14, 32, 33]. By introducing accessory genes that give benefits like virulence, environmental adaptability, or resistance to antibiotics, prophages, phage genomes integrated into bacterial chromosomes, can have a substantial impact on bacterial fitness [33, 34]. Prophages are a possible vector for the spread of virulence factors and ARGs among various ecological niches, such as human, animal, and environmental reservoirs, in the context of ESBL-EC [33, 35]. By highlighting source-specific variations in prophage features and their possible public health implications, the findings presented here expand on our current understanding of prophage biology and ecological roles. The present study incorporates these findings to address the wider relevance of prophages in relation to the spread of ESBL-EC and the One Health paradigm, which acknowledges the interdependence of human, animal and environmental health [36, 37].

One study investigated the diversity of phages isolated from ESBL-EC strains originating from poultry in Nigeria [17]. Different ecological and environmental factors that affect phage diversity and abundance are reflected in the dispersion of phages from various sources [16, 32, 33, 38]. Our study detected intact phages across the different ESBL-EC strain sources. Intact phages influence phage dynamics, but new infections occur only when environmental conditions induce the lytic cycle leading to host lysis and the release of phage particles [14, 32, 39]. Compared to human and cattle samples, environmental samples probably face less selective pressure, which results in less variation in phage populations. Environmental factors including temperature, nutrition availability, and rival microbial communities, however, may still have an impact on phage diversity and abundance in these environments [32, 40, 41].

The results of the present study are consistent with earlier research showing that higher bacterial numbers and selective pressures make host-associated environments, such as the microbiomes of humans and animals, hotspots for phage activity [42, 43]. On the other hand, although being more stable, environmental samples might act as phage diversity repositories that add to the gene pool through horizontal gene transfer events [41].

These results highlight the necessity of integrated, source-specific analyses to gain a deeper understanding of the function of phages in microbial ecosystems and offer a framework for examining the interactions between phage populations and their surroundings [14, 32, 41].

Our results highlight how crucial genome size is as a measure of phage adaptation and diversity. Greater genetic complexity is frequently reflected in larger genomes, which may improve the phage's capacity to infect a wider variety of bacterial hosts or endure in changing environmental circumstances [44, 45]. On the other hand, phages with smaller genomes might be more streamlined and tailored to particular ecological niches [38]. To gain a better understanding of the factors influencing genome size variability, more research is necessary to examine the genetic makeup and functional capacities of phages derived from various sources.

This study identified both temperate and lytic intact phages within the *Caudoviricetes* class and *Peduoviridae* family. The lytic phages detected were specifically observed in the *Punavirus* genus of the *Caudoviricetes* class, as they are predominantly lytic and serve as effective tools for controlling bacterial populations [17, 46, 47]. *Peduoviridae* phages are primarily temperate aiding in bacterial evolution and horizontal gene transfer supporting our results [46, 47]. Prior reports of this and other phage families in humans, animals, and the environment have been made [17, 46, 48].

Patterns of microbial ecology and possible transmission dynamics are suggested by the observed distribution of phage host genera across various sources in the present study. *Escherichia*'s prevalence in the microbiomes of humans and cattle is consistent with its supremacy as a host genus [3, 49]. Additionally, the presence of *Salmonella* and *Edwardsiella* suggests potential zoonotic transmission pathways [50]. These findings underscore the importance of phage-host interactions in shaping microbial populations across different ecosystems.

The present study detected phage associated ARGs, ESBL genes, virulence genes and heavy metal resistance in ESBL-EC isolates originating from abattoir workers, beef cattle and the abattoir environment. The fact that intact phages from human, animal, and environmental samples contain these genes demonstrate how widespread these genetic elements are and how bacteriophages aid in their spread. These results highlight the significance of considering intact phages as possible vectors and reservoirs for the horizontal spread of virulence factors and AMR in various ecosystems. According to several studies, bacteriophages act as carriers of genes in meat and should not be underestimated as contributors to the global antibiotic resistance epidemic [51, 52]. Phages have also been reported as reservoirs of ARGs in aquatic environments further supporting our results [53]. These findings further support the claims of our study. In contrast, other studies including a recent Nigerian study have reported that ARGs are rarely detected in phages [17, 54].

The detection of the phage-associated heavy metal resistance gene *emrE* in ESBL-EC isolates from abattoir workers, beef cattle and abattoir environment suggest a widespread capacity for resistance to heavy metal stress at the one health interface. The *emrE gene* in *E. coli* encodes a multidrug efflux pump associated with resistance to various toxic compounds, including disinfectants, antibiotics, and heavy metals [55]. This finding highlights the potential for these isolates to survive in environments contaminated with heavy metals, such as industrial or agricultural sites [56]. It also raises concerns about the co-selection of AMR, as heavy metal resistance genes are often located on the same mobile genetic elements as AMR genes, potentially exacerbating the spread of resistance [56].

Phage-like plasmids are hybrid mobile genetic elements that have the capacity to contribute to the dissemination of ARGs using the three known HGT mechanisms. The detection of these elements carrying ARGs encoding tetracycline (*tet(A)* and ESBL (*bla*_*CTX-M-55*_) in human-associated isolates suggests that humans could play a significant role in the dissemination of these ARG genes in the meat production settings. The discovery of phage-associated plasmids in ESBL-EC from both human and cattle sources highlights the function of bacteriophages in promoting horizontal gene transfer of AMR genes across various reservoirs. These plasmids, which include bacteriophage sequences, have been linked to the propagation of virulence factors and AMR, which may increase the pathogenicity and survival of bacterial strains [57, 58]. Concerns regarding the zoonotic potential of such resistant strains are raised by the discovery of phage-associated plasmids in both abattoir workers and beef cattle isolates, which points to a shared genetic pool between these species.

The identification of a tail spike protein in bacterial isolates from humans and cattle points to a possible route for bacteriophage-mediated horizontal gene transfer. These proteins, which are usually linked to bacteriophage structures, have been linked to making phage attachment and infection easier, which may help in genetic exchange between species [59, 60]. The protein's high frequency in isolates from both humans and cattle raises serious questions about the bacterial strains' propensity to spread disease and suggests that human and animal populations may share reservoirs [59, 60]. The discovery of these proteins highlights the necessity of surveillance in veterinary and clinical settings, especially for infections that can spread across species, considering the One Health perspective.

Investigating alternate tactics to fight multidrug-resistant bacteria, such as ESBL-EC, has become necessary due to the growing threat of AMR on a worldwide scale [1, 2]. Consequently, bacteriophages present a promising and new approach in this context since they provide highly specific, targeted mechanisms for eliminating bacterial infections while reducing collateral damage to the microbiota [14, 38, 61]. The observed high abundance and variability of total phages across abattoir workers, beef cattle, and the abattoir environment underscore the dynamic interplay between bacterial hosts and environmental factors, shaping phage ecology [62]. The notable variation in intact phages, particularly in ESBL-EC from humans and cattle, suggests potential host-specific selective pressures driving phage diversity. The parallels and discrepancies in phage genome sizes observed in ESBL-EC originating from abattoir workers, beef cattle, and the abattoir environment further highlight the complexity of phage-host interactions and possible niche adaptations. The predominance of *Peduoviridae* and *Punavirus* genus [17, 46, 47], along with the frequent detection of key phage-associated genes (e.g., integrase, capsid, lysin), supports the idea that horizontal gene transfer plays a significant role in shaping bacterial and phage communities [56, 57, 63]. The high-confidence phage-host associations, particularly the prevalence of *Escherichia* spp. in both human and beef cattle microbiomes, reinforce the interconnected nature of microbial ecosystems [3, 49].

From a One Health and climate perspective, the detection of ARGs, ESBL genes, virulence genes, heavy metal resistance elements and plasmids in bacteriophages raises concerns about their potential role in the dissemination of AMR under changing environmental conditions. Climate variability—through alterations in temperature, precipitation, and nutrient availability—could impact bacterial host populations, consequently influencing phage dynamics and AMR gene transfer [64, 65]. Additionally, the detection of tail spike proteins in phages from both abattoir workers and beef cattle in a shared abattoir environment suggests possible cross-species transmission, raising questions about the broader ecological impact of bacteriophages in maintaining or disrupting microbial homeostasis [66]. Taken together, these findings suggest that environmental shifts, driven by climate change, could impose selective pressures that influence phage-host dynamics, microbial fitness costs, and the persistence of resistance and virulence determinants in microbial ecosystems [67, 68].

There are numerous applications for phages in the fight against AMR. The first is phage treatment, which involves using lytic phages directly to target and eradicate bacteria that are resistant to antibiotics, such ESBL-EC, using methods other than those found in conventional antibiotics [69, 70]. Phage-based approaches may be crucial in One Health's efforts to reduce the spread of ESBL-EC in human, animal, and environmental reservoirs [36, 37]. The burden of resistant bacteria entering the human population may be lessened by their use in wastewater treatment, veterinary medicine, and agriculture. Phage treatment has a lot of potential to stop the spread of resistance and maintain the effectiveness of current antibiotics when combined with genomic surveillance in AMR control measures. Phage-based medicines, which provide targeted, flexible, and environmentally friendly ways to address the increasing burden of ESBL-EC, are ultimately a game-changing weapon in the fight against AMR [69–71].

The results of this present study and their generalizability may have been influenced by the limitations of this investigation. Unequal sampling across sources and potential selection bias, present notable constraints. Specifically, one of the abattoirs had a low participation rate, which reduced the sample size from this source and might have potentially skewed our findings. Furthermore, not all host types had the same level of ESBL-EC detection; environmental samples had the least detection possibly due to their smaller sample size when compared to abattoir workers and beef cattle. Additionally, selection bias might have been introduced by the study's reliance on voluntary participation of abattoir workers, especially in settings with low participation. These limitations highlight the need for larger, more representative datasets and more thorough sampling techniques to strengthen the study’s conclusions.

## Conclusions

This study provides critical insights into the diversity, distribution, and genomic characteristics of prophages integrated within ESBL-EC originating from abattoir workers, beef cattle and abattoir environment in Nigeria. Our results emphasize the intricate and interrelated dynamics of bacteriophages in human, animal, and environmental reservoirs, highlighting their function in promoting gene transfer and forming microbial communities. The diversity of intact phages, especially in ESBL-EC, indicate that environmental variables and bacterial host populations are important determinants of phage diversity. The functional role of phages in microbial evolution is highlighted by the dominance of *Peduoviridae* and *Punavirus* genus as well as the presence of genes involved in integration, structural assembly, and host interaction. Furthermore, the detection of heavy metal resistance genes, virulence factors, plasmids and ARGs within phage genomes raises important questions regarding their possible role in the persistence and spread of these elements across various environments through horizontal gene transfer.

To capture temporal and regional trends in ESBL-EC and prophage dynamics, future research should try to use more representative and longitudinal sampling. Understanding phage-host interactions from a One Health perspective is essential for anticipating microbial adaptations and preventing the spread of resistance considering the growing environmental constraints brought on by climate change. The ecological and evolutionary ramifications of phage-mediated gene transfer should be investigated in future research, especially considering anthropogenic effects and environmental disturbances.

## Supplementary Information

Supplementary Material 1.

## Data Availability

This Whole-Genome Shotgun project has been deposited at DDBJ/ENA/GenBank under the BioProject accession PRJNA797451. All data generated or analysed during this study are included in this published article [and its supplementary information files].

## Abbreviations

AMR: Antimicrobial resistance
CARD: Comprehensive Antibiotic Resistance Database
ESBL-EC: Extended-spectrum beta-lactamase-producing *E. coli*
ARGs: Antibiotic resistance genes
HGT: Horizontal gene transfer
iTOL: Interactive Tree of Life
VFDB: Virulence Factor Database
